# Physical Activity, Ability to Walk, Weight Status, and Multimorbidity Levels in Older Spanish People: The National Health Survey (2009–2017)

**DOI:** 10.3390/ijerph17124333

**Published:** 2020-06-17

**Authors:** Pedro Ángel Latorre-Román, Juan Manuel Carmona-Torres, Ana Isabel Cobo-Cuenca, José Alberto Laredo-Aguilera

**Affiliations:** 1 Department of Didactics of Corporal Expression, University of Jaén, Campus Las Lagunillas s/n, 23071 Jaén, Spain; platorre@ujaen.es; 2 Facultad de Fisioterapia y Enfermería, Universidad de Castilla-La Mancha, Campus de Fábrica de Armas, Av de Carlos III, 45071 Toledo, Spain; anaisabel.cobo@uclm.es; 3 Grupo de Investigación Multidisciplinar en Cuidados (IMCU), Campus de Fábrica de Armas, Universidad de Castilla-La Mancha, Av de Carlos III, 45071 Toledo, Spain; josealberto.laredo@uclm.es; 4 Facultad de Ciencias de la Salud, Universidad de Castilla-La Mancha, Av Real Fábrica de Sedas s/n, 45600 Talavera de la Reina, Spain

**Keywords:** older people, physical activity, weight status, gait, aging

## Abstract

Background. Many studies have shown a relationship between physical functioning and health status in older people. Aim. The purpose of this study was to analyze the temporal trends of physical activity (PA), ability to walk, weight status, self-perceived health, and disease or chronic health problems in people over 65 years from 2009 to 2017, using the European Health Survey in Spain and the National Health Survey in Spain. Methods. This study included 13,049 older people: 6026 (2330 men and 3696 women; age (mean, SD (Standard Deviation)) = 75.61 ± 7.11 years old) in 2009 and 7023 (2850 men and 4173 women; age (mean, SD) = 76.01 ± 7.57 years old) in 2017. Results. In 2017, older people exhibited lower values of moderate PA (*p* < 0.001), a lower number of hours of walking per week (*p* < 0.001), and worse self-perceived health status (*p* < 0.001) compared to 2009. These differences are maintained when comparing the sexes. Compliance with PA recommendations was 27.9% and 6.1% (chi-squared = 352.991, *p* < 0.001) in 2009 and 2017, respectively. There were no significant differences in weight status between older people in 2009 and 2017. In 2017, older people had significantly high percentages of disease or chronic health problems (*p* < 0.05), number of diseases (*p* < 0.001), severe difficulty walking 500 m without assistance (*p* < 0.05), and severe difficulty going up or down 12 stairs. Conclusions. From 2009 to 2017, Spanish older people worsened their PA levels and perception of their health status, and they increased their disease levels, which could be associated with the worsening of ability to walk in 2017.

## 1. Introduction

The global population aged 60 years or over numbered 962 million in 2017, and it is expected to double by 2050, when it is projected to reach nearly 2.1 billion. In 2017, in Spain, 25.3% (11.75 million) of people were aged 60 years or over [1]. This has promoted a change in the management of society, with a greater focus on older people due to the increase in life expectancy [2]. In Spain, the life expectancy in 2018 was 85.9 years for women and 80.9 years for men [3].

Chronic diseases and functional impairment are the most important health determinants in older adults [4,5]. Thereby, the prevalence of multimorbidity increases significantly with age [6]. With aging, older people walk slower, have less muscle strength, have poorer memory and reasoning abilities, and are slower to respond in cognitive tasks relative to younger adults and to themselves when they were younger; these manifestations are the result of neural cell loss in the frontal, parietal, and temporal lobes, and they strongly depend on hypofunction of the monoaminergic and cholinergic pathways [7,8]. Therefore, aging has been associated with frailty and functional limitations due to three factors: an irreversible biological process, deconditioning due to a sedentary lifestyle, and comorbidity effects [9].

In addition, along with aging, there is an increase in sensibility to external aggressions that cause fragility, deterioration of the functional reserve, sarcopenia, disability, falls, and hospitalization, thus reducing the quality of life [10] and physical fitness [11]. These decrements have been associated with increased incidence of type 2 diabetes [12], cardiovascular disease [13], and risk of falls [14]. Therefore, multimorbidity in older people shows a significant negative impact on health-related quality of life [15].

Moreover, the lack of physical activity (PA) that predominates in older people causes premature onset of ill health, disease, and frailty [16]. In this sense, physical inactivity has been identified as the fourth leading risk factor for global mortality (6% of deaths globally) [17]. Sedentary behavior and PA are important factors in the lifestyle of people, which have an influence on body composition [18] and detrimental associations with sarcopenia and muscle mass [19] in aged adults. In this regard, in older people, the highest levels of functional limitations are associated with obesity and overweight when compared to people with normal weight, regardless of the level of PA [20].

PA is a very promising non-pharmacological method for the promotion of health and is available to all people [21]. In older people, moderate PA has a positive effect in preventing coronary heart disease, reduces mortality, prevents type 2 diabetes and stroke, reduces blood pressure, reduces the development of dementia and falls, and improves the quality of life [22,23,24,25].

Therefore, PA is the main indicator of health in the older people. In particular, walking performance is an especially strong biomarker of health [26]. Social ambulation requires the ability to adapt walking characteristics to environmental demands [27]. In this regard, performance in daily activities, including walking through obstacles, going up a curb, climbing stairs, multi-surface terrains, etc., are associated with executive function [28,29]. Older people who report higher levels of disease or chronic health problems show severe difficulty walking 500 m without assistance and going up and down 12 stairs [30]. In turn, the preferred walking speed in older people is an indicator of general health and survival, and safe walking requires intact cognition and executive control [31].

Because PA has important health benefits in old age, it is necessary to measure the trends of PA in older people. Although objective tests such as the portable accelerometer are the most reliable methods of measuring PA, in older people, questionnaires such as the international physical activity questionnaire (IPAQ) are also very suitable methods [32]. Particularly, the short version of IPAQ can be applied in local and national studies where PA monitoring is required [33].

From a public health perspective, taking the above information into account, the purpose of this study was to analyze the temporal trends of PA, ability to walk, weight status, self-perceived health status, and disease or chronic health problems in people over 65 years from 2009 to 2017, using the European Health Survey in Spain (EHSS) and the National Health Survey in Spain (NHSS).

## 2. Materials and Methods

### 2.1. Participants

In a cross-sectional study, we used secondary data from the EHSS 2009 [34] and the NHSS 2017 [35] through self-reported information. Personal interviews of EHSS and NHSS were conducted by the INE and MSSI using a probabilistic multistage sampling with stratification of the first-stage units (census sections) and the second-stage units (main family dwellings), with the final units (individuals) being selected by means of random routes and sex- and age-based quotas. The NHSS has national and autonomous representativeness. The study population was restricted to non-institutionalized population over 65 years of age from urban and rural areas, residing in family dwellings. The exclusion criteria were age under 65 years and inability to respond to the interview, whether due to disability, illness, ignorance of the language or any other barrier. This study included 13,049 older people: 6026 (2330 men and 3696 women; age (mean, SD) =75.61 ± 7.11 years old) in 2009 and 7023 (2850 men and 4173 women; age (mean, SD) = 76.01 ± 7.57 years old) in 2017. For data analysis, the sample was divided into three age groups: 65–74, 75–84, and ≥85 years old. The data obtained from the surveys are extracted from the National Statistics Institute and the Ministry of Health, Social Services and Equality web pages in the form of anonymized microdata, so no authorization is required for their use. According to Spanish law, no report from the ethics committee is required to use anonymous and public data from these institutions.

### 2.2. Material and Testing

The data collection instruments used by the INE and MSSI, in a transversal way, were the 2009 and 2017 EHSS [34,35,36]. In this survey, participants are asked about their sociodemographic variables, PA, ability to walk, health self-perception, disease or chronic/long-lasting health problem, and anthropometric characteristics such as body mass, height, and BMI: weight (kg)/height (cm)^2^. The BMI was categorized according to the World Health Organization (WHO) criteria (< 18.5 kg/m^2^, underweight; 18.5–24.9 kg/m^2^, normal; 25.0–29.9 kg/m^2,^ overweight; ≥ 30 kg/m^2^, obese) [37].

PA was measured by short version of the IPAQ, Spanish version [32]. PA was defined as the level of self-reported engagement in moderate activity in a typical week: both days/week and h/week and walking for 10 min (days/week). Moderate PA was defined as PA that causes breathing somewhat more heavily than normal and may include carrying light weights, riding a bicycle at normal speed, sports, or gardening. In addition, fitness was assessed by two types of functional outcomes or functional limitations: the ability to walk 500 m without assistance and to go up and down 12 stairs. Finally, in accordance with the WHO [6], multimorbidity was considered as the coexistence of two or more chronic conditions in the same individual, and data were collected by medical diagnosis. A total of 32 diseases were diagnosed. Health self-perception was evaluated with a Likert-score question, with responses ranging from 1 to 5 (very good to very bad).

### 2.3. Statistical Analysis

Data were analyzed using SPSS v.24.0 for Windows (SPSS Inc, Chicago, IL, USA), licensed to the University of Castilla-La Mancha. The significance level was set at *p* < 0.05. Descriptive data are reported in terms of means, standard deviations (SD), and percentages. The Mann–Whitney U test was used to compare non-continuous variables between groups (2009 vs. 2017 and male vs. female); for continuous variables, analysis of variance (ANOVA) adjusted by the Bonferroni test was employed. The chi-squared test was used to compare nominal variables between groups (2009 vs. 2017 and male vs. female). Pearson correlation analysis was conducted between the numbers of diseases (multimorbidity levels), BMI, PA, age, and health self-perception. The multimorbidity levels threshold that best discriminated several difficulties in the ability to walk 500 m without assistance and to go up and down 12 stairs was determined by using the receiver operating characteristic (ROC) curve.

## 3. Results

The sociodemographic variables showed that 49.6% of participants completed primary schooling, 49.8 % were married, and 14% were unskilled workers. Table 1 shows age, anthropometric variables, PA, and self-perceived health status regarding sex and survey year. The mean age increased significantly between 2009 and 2017, from 75.61 ± 7.11 to 76.01 ± 7.57 years for the whole sample (*p* = 0.002), from 74.83 ± 6.87 to 74.85 ± 7.07 years (*p* ≥ 0.05) for males, and from 76.11 ± 7.21 to 76.81 ± 7.79 years for females (*p* < 0.001). The population over age 90 increased significantly from 2009 to 2017 (*n* (%) = 192 (3.2) vs. 331 (4.7) Δ1.5%, *p* < 0.05, respectively). Regarding weight status, women displayed significantly lower values for BMI in 2017 than in 2009. Moreover, across the whole sample, in 2017 elderly people exhibited lower values of moderate PA (*p* < 0.001), a lower number of hours of walking per week (*p* < 0.001), and worse self-perceived health status (*p* < 0.001) compared to 2009. These differences are maintained when comparing the sexes.

In the whole sample, compliance with PA recommendations was 27.9% and 6.1% (chi-squared = 352.991, *p* < 0.001) in 2009 and 2017, respectively. In addition, significant differences were found in both women (28.9% vs. 4.8%, chi-squared = 234.199, *p* < 0.001) and men (26.4% vs. 7.7%, chi-squared = 118.937, *p* < 0.001) in 2009 and 2017, respectively.

**Figure 1:**
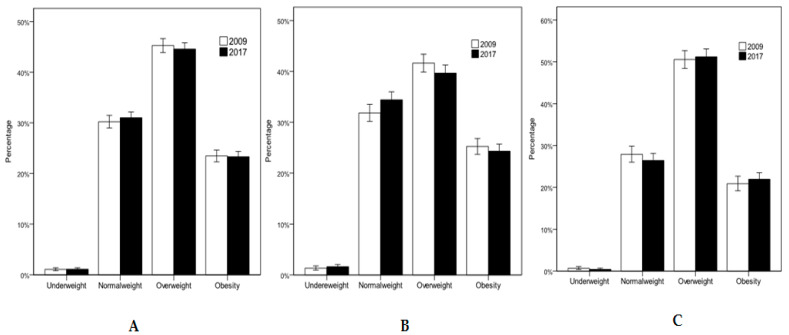
Weight status in the total sample (**A**), in women (**B**), and in men (**C**).

Weight status is shown in Figure 1. There were no significant differences between older people in 2009 and 2017. The overweight and obesity prevalence in 2009 and 2017 was 45.3%/23.4% and 44.6%/23.3%, respectively. In men, the overweight and obesity prevalence in 2009 and 2017 was 50.5%/20.9% and 51.2%/21.9%, respectively. In women, the overweight and obesity prevalence in 2009 and 2017 was 41.6%/25.2% and 39.7%/24.3%, respectively.

Table 2 shows the disease or chronic health problems and ability to walk regarding survey year and sex. In whole group, 2017 showed significantly higher percentages of disease or chronic health problems (*p* < 0.05), number of diseases (*p* < 0.001), severe difficulty walking 500 m without assistance (*p* < 0.05), and severe difficulty going up or down 12 stairs than 2009. These results were similar for both women and men comparing 2009 to 2017.

**Figure 2:**
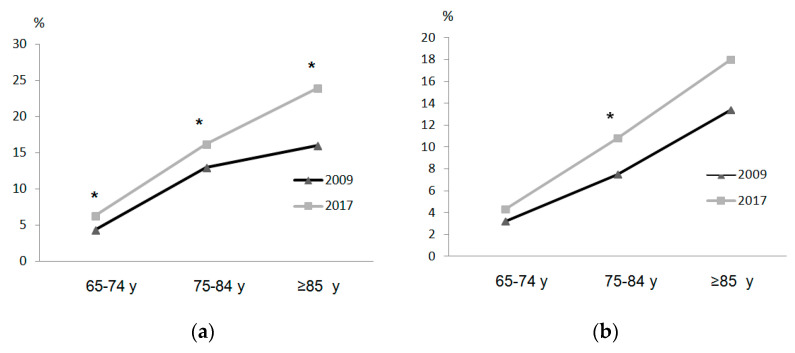
Difficulty walking 500 m without assistance: women (**a**) and men (**b**). * *p* < 0.05.

**Figure 3:**
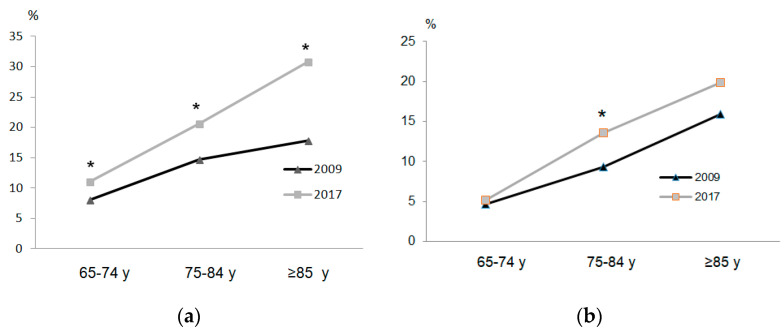
Difficulty going up or down 12 stairs: women (**a**) and men (**b**). * *p* < 0.05.

The disease or chronic health problems and ability to walk regarding survey year, sex, and age groups are shown in Table 3 and Figure 2. In all age groups in 2017, both women and men had higher percentages of disease or chronic health problems and number of diseases than in 2009. Likewise, in 2017 there was a general deterioration of self-perceived health status regardless of sex and age group. In response to questions (Figure 2 and Figure 3) regarding severe difficulty walking 500 m without assistance and difficulty going up or down 12 stairs, women in all age groups in 2017 displayed greater percentages of severe difficulty than groups in 2009. However, in men, there were significant differences in the 75–84-year group.

**Figure 4:**
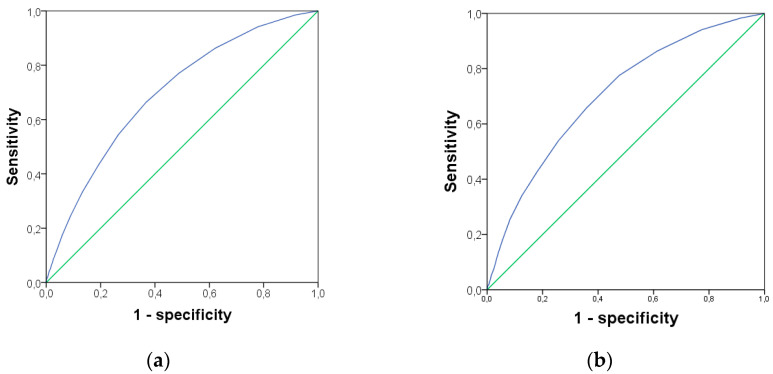
ROC curve that summarizes the potential of number of diseases to identify severe difficulty walking 500 m without assistance (**a**) and severe difficulty going up or down 12 stairs (**b**).

The multimorbidity showed a significant increase from 2009 to 2017 at 71.9% vs. 85.9% (*p* < 0.001), respectively. Figure 4 shows the ROC curve for severe difficulties in the ability to walk 500 m without assistance (left) and to go up and down 12 stairs (right) predicted by the number of diseases; area under the curve (AUC) = 0.697, 95% CI = 0.683–0.712; *p* < 0.001, the cut point was 4.5 diseases (sensitivity = 0.663, 1-specificity = 0.367) and AUC = 0.704, 95% CI = 0.691–0.717; *p* < 0.001, the cut point was 4.5 diseases (sensitivity = 0.657, 1-specificity = 0.357) respectively (Figure 4).

Finally, in the whole sample, Pearson correlation analysis revealed a significant correlation among the multimorbidity levels and age (r = 0.144; *p* < 0.001), BMI (r = 0.159; *p* < 0.001), and self-perceived health (r = 0.480; *p* < 0.001).

## 4. Discussion

The purpose of this study was to analyze PA, weight status, self-perceived health status, and ability to walk in older people aged 65 and over using the EHSS conducted in the period 2009–2017. The main findings of this study were that older people in the year 2017 showed a significant reduction of moderate PA and self-perceived health status, higher percentages of disease or chronic health problems, and, in consequence, higher percentages of functional limitations than people in the year 2009. However, there were no significant changes regarding weight status.

The current study shows how moderate PA and the ability to walk were reduced from 2009 to 2017. The WHO offers recommendations for performing moderate PA for at least 150 min, 75 min of vigorous PA, or a combination of both during the week [21]. In this study, both women and men in the 2017 group did not achieve the physical recommendations in relation to moderate PA levels. The current study shows how moderate PA and the weekly hours of walking were reduced from 2009 to 2017.

In accordance with the present results, a previous study demonstrated that through objective measurement of sedentary behavior, older adults spend an average of 9.4 h a day sedentary (65–80% of their waking day). In this regard, there is an association between sedentary lifestyle and aging [38]. Moreover, the results of the current study match those observed in an earlier study in the Spanish senior population in 2013 (65–75 years), noting a high percentage of inactive senior people regarding moderate PA; however, the percentage of the sample who did not meet international PA recommendations was lower (39.3%) than the results obtained in this study for both 2009 and 2017 [39]. These findings confirm the trend towards a decrease in the prevalence of PA and the ability to walk, from 2006 to 2011, in Spanish people over 65 years [40]. Furthermore, in both sexes, being over 80 years of age and having a poorer perception of health and some type of disability were significantly related to a decrease in PA [40]. However, the trend of PA reduction over time found in this study does not support previous research of PA time trends conducted in developed countries indicate that PA levels appear to be increasing [41].

As previously mentioned, walking performance in older people is considered a strong health biomarker [26]. Recently, it was noted that walking speed (≥8 m/s) modifies the prognosis of cardiovascular disease, neuropsychiatric multimorbidity, and mortality in older people [4]. In addition, the inability and time to travel a 400-m walk is a predictor of mortality in older people [42]. In the current study, older people reported low percentages of severe difficulties in walking 500 m without assistance and climbing 12 stairs, in both 2009 and 2017. However, these functional limitations were higher in 2017 than 2009 and increased in all age groups. Therefore, these results may be related to increase in multimorbidity levels and reduction of PA in 2017. Our findings add to past research that showed that in adults ≥ 50 years old, physical function is related to the development and worsening of multimorbidity over time [43]. Likewise, a previous study showed this trend, where the prevalence of multimorbidity increased from 38.2% in 2006–2007 to 41.5% in 2015 in older adults of 10 European health systems [44]. Conversely, other research showed that the worst functional status in older people appear to be independent of PA level [45].

Another possible explanation for this could be that ability to walk is influenced not only by PA; furthermore, muscle strength and muscle mass are related to aging. In this sense, maximal voluntary strength production decreases with age and contributes to physical dependence and mortality [46]. Moreover, the low percentages of functional limitations in this population can be explained because both 2009 and 2017, low percentages of obesity were found in relation to other studies [47]. In this regard, the cut-off point BMI to increase the risk of disability in the older people may be greater than 30 kg/m^2^ [48]. In the present work, both in 2009 and 2017, BMI values were below this cut-off point. In this sense, obese older people have a significantly higher risk of mobility limitation compared to non-obese people, regardless of lifestyle and other factors [49]. Therefore, there is an inverse dose–response association between PA levels and multimorbidity [50] and multimorbidity and increases of functional limitations in older people [51]. In addition, psychological factors such as depression are associated with lower physical activity [52]. However, depression was not discussed in the current study. Broadly, more research is needed, through longitudinal follow-up studies, to better understand cause-effect relationship between PA, ability to walk, weight status, and multimorbidity levels in older people.

Finally, some limitations in this study must be mentioned. The main limitation is the cross-sectional design, so caution must be exercised when interpreting the observed associations. Another limitation is the measurement of PA levels, because it is self-reported and without objective measures. For their registration, participants confirmed their PA levels in the last seven days according to the short version of the IPAQ validated in 12 countries. Another limitation of this study is that other sociodemographic determinants and the heterogeneous characteristics of the sample might have influenced in these results. For future studies, objective measurement of the levels of PA is recommended as well as the measurement of body composition in order to analyze associations between PA, body composition, morbidity, and ability to walk, which can provide more information about the results of the study. The present work has great strength in the sample obtained and its distribution throughout the country, making it nationally representative.

## 5. Conclusions

In conclusion, from 2009 to 2017, Spanish older people worsened their PA levels and the perception of their health status, and they increased their disease levels, which could be associated with the worsening of ability to walk in 2017. However, they did not experience an increase in overweight and obesity prevalence. The current data highlight the importance of incorporating exercise programs at an early stage of aging in order to preserve physical performance and to prevent negative consequences of aging related to health status and disease prevalence.

## Figures and Tables

**Table 1:** Age, anthropometric variables, physical activity, and self-perceived health status regarding survey year and sex (mean ± SD (Standard Deviation)).

Characteristics	2009		2017		*p*-Value	2009				2017			
	*n*	All	*n*	All		*n*	Male	*n*	Female	*n*	Male	*n*	Female
Age (years)	6026	75.61 (7.11)	7023	76.01 (7.57)	0.002	2330	74.83 (6.87)	3696	76.11 (7.21)	2850	74.85 (7.07)	4173	76.81 (7.79) ***
Body mass (kg)	5110	71.61 (12.43)	6371	72.13 (13.06)	0.031	2103	77.03 (11.70)	3007	67.81 (11.49)	2717	78.49 (11.89) ***	3654	67.39 (11.82)
Body height (cm)	5110	162.02 (8.49)	6371	162.71 (8.42)	<0.001	2103	168.36 (6.92)	3007	157.67 (6.47)	2717	169.23 (6.54) ***	3654	157.86 (6.51)
BMI (kg/m^2^)	5110	27.25 (4.27)	6371	27.21 (4.39)	0.662	2103	27.17 (3.73)	3007	27.31 (4.61)	2717	27.39 (3.83)	3654	27.08 (4.76) *
To walk (h/week) ≠	2133	7.11 (17.12)	1372	1.28 (1.00)	<0.001	958	7.88 (5.99)	1175	6.49 (22.41)	626	1.43 (1.10) ***	746	1.15 (0.90) ***
Moderate PA (h/week) ≠	2588	3.75 (6.67)	405	1.32 (1.11)	<0.001	1101	2.94 (5.70)	1487	4.36 (7.25)	184	1.52 (1.34) **	221	1.14 (0.84) ***
Moderate PA (days/week) ǂ	2600	2.16 (2.96)	1704	0.94 (1.96)	<0.001	1108	1.91 (2.84)	1492	2.35 (3.04)	778	1.10 (2.19) ***	926	0.82 (1.72) ***
Self-perceived health status (1–5) ǂ	6026	2.86 (0.96)	7023	2.70 (0.92)	<0.001	2330	2.68 (0.91)	3696	2.97 (0.97)	2850	2.56 (0.87) ***	4173	2.79 (0.93) ***

* *p* < 0.05, ** *p* < 0.01, *** *p* < 0.001 in relation to sex group in 2009; **ǂ** Mann–Whitney U test was performed; ≠ Only the results of the group of 65–74-year-olds.

**Table 2:** Disease or chronic health problems and ability to walk regarding survey year and sex.

Characteristics		2009	2017	2009		2017	
		All	All	Male	Female	Male	Female
Disease or chronic/long-lasting health problem (≥6 month) *n* (%)	Yes	5028 (83.4)	6493 (92.5) *	1848 (79.3)	3180 (86.0)	2585 (90.7) *	3908 (93.6) *
Difficulty walking 500 m without assistance *n* (%)	Severe difficulty	490 (8.1)	786 (11.2) *	136 (5.8)	354 (9.6)	231 (8.1) *	555 (13.3) *
Difficulty going up or down 12 stairs *n* (%)	Severe difficulty	626 (10.4)	1041 (14.8) *	177 (7.6)	449 (12.1)	278 (9.8) *	763 (18.3) *
Number of diseases; mean (SD)		3.27 (2.55)	5.06 (3.46) ***	2.54 (2.14)	3.73 (2.68)	4.29 (3.01) ***	5.58 (3.64) ***

* *p* < 0.05, *** *p* < 0.001, indicate significant differences in relation to whole sample, male or female, in 2009.

**Table 3:** Age, BMI, disease or chronic health problems, number of diseases, and self-perceived health status regarding survey year, age group, and sex.

Characteristics	2009		2017		2009		2017		2009		2017	
	65–74 Years		65–74 Years		75–84 Years		75–84 Years		≥85 Years		≥85 Years	
	Male *n* = 1206	Female *n* = 1642	Male *n* = 1549	Female *n* = 1834	Male *n* = 890	Female *n* = 1537	Male *n* = 979	Female *n* = 1564	Male *n* = 232	Female *n* = 519	Male *n* = 322	Female *n* = 775
Age (year) mean (SD)	69.37 (2.97)	69.57 (3.01)	69.41 (2.77)	69.46 (2.83)	78.78 (2.72)	78.95 (2.77)	79.13 (2.92) **	79.60 (2.80) ***	88.09 (3.03)	88.35 (3.21)	87.99 (2.90)	88.55 (3.31)
BMI (kg/m^2^) mean (SD)	27.50 (3.71)	27.53 (4.61)	27.77 (4.00)	27.22 (4.83) *	26.98 (3.71)	27.44 (4.60)	27.18 (3.55)	27.26 (4.66)	25.96 (3.66)	26.05 (4.47)	26.04 (3.41)	26.29 (4.75)
Disease or chronic/long-lasting health problems (≥6month) Yes (%)	915 (75.7)	1356 (82.7)	1367 (88.3) ***	1664 (90.7) ***	737 (82.8)	1361 (88.5)	917 (93.7) ***	1497 (95.7) ***	196 (84.5)	463 (89.2)	301 (93.5) **	747 (96.4) ***
Number of diseases; mean (SD)	2.26 (1.96)	3.61 (2.75)	3.87 (2.91) ***	4.95 (3.54) ***	2.74 (2.18)	3.83 (2.64)	4.69 (3.01) ***	6.16 (3.71) ***	3.20 (2.57)	3.80 (2.54)	5.09 (3.17) ***	5.59 (3.48) ***
Self-perceived health status (1–5) ǂ mean (SD)	2.54 (0.87)	2.84 (0.97)	2.41 (0.82) ***	2.60 (0.89) ***	2.78 (0.92)	3.05 (0.97)	2.73 (0.88)	2.92 (0.92) ***	3.01 (0.97)	3.12 (0.97)	2.82 (0.92) *	2.97 (0.96) **

* *p* < 0.05, ** *p* < 0.01, *** *p*< 0.001, significant differences in relation to 2009. **ǂ** Mann–Whitney U test was performed.
